# Straight or curved? From deterministic to probabilistic models of 3D motion perception

**DOI:** 10.3389/fnbeh.2013.00079

**Published:** 2013-07-05

**Authors:** Martin Lages

**Affiliations:** School of Psychology, University of GlasgowGlasgow, UK

The perceptual inference of the three-dimensional (3D) external world from two-dimensional (2D) retinal input of the left and right eye is a fundamental problem (Berkeley, 1709/1975; von Helmholtz, 1910/1962) that the visual system has to solve through neural computation (Poggio et al., 1985; Pizlo, 2001). This is true for static scenes as well as for dynamic events. The inverse problem for binocular 3D motion perception implies that the visual system estimates object motion in 3D space from spatial-temporal processing of binocular input. How exactly the visual system achieves these representations is not well understood (Harris et al., 2008).

Velocity in 3D space is typically described by motion direction and speed. In computer vision local velocities are conveniently expressed as vectors in a 3D co-ordinate system. Establishing motion vectors is desirable because local estimates in a sufficiently dense vector field can provide the basis for segmenting an object from its background, for identifying an object, and for planning and executing actions in a dynamic environment. However, a “straight” or linear vector representation can be problematic when trying to capture the perception of curved or non-linear motion.

In the study by Pierce et al. (2013) observers discriminated curved (convex and concave) from straight (linear) motion trajectories of an object moving horizontally through depth. Although human discrimination performance may depend on subtle aspects of the viewing geometry, smooth pursuit, stimulus characteristics and accommodation cues of individual observers (Nefs et al., 2010; Heron and Lages, 2012) the results by Pierce et al. clearly suggest that binocular input facilitates the detection of curved trajectories in depth.

Interocular velocity difference (IOVD) or changing disparity over time (CDOT) are both binocular inputs that may have improved discrimination performance in this task. However, motion detection and therefore IOVD is relatively insensitive to velocity changes (Gottsdanker, 1956; Calderone and Kaiser, 1989; Lisberger and Movshon, 1999) whereas disparity processing and therefore CDOT needs to be coupled with spatio-temporal information to discern a 3D motion trajectory. As a consequence deterministic models of motion perception, such as IOVD and CDOT, may be too limited to match the wealth of perceived motions. It also seems unlikely that the visual system has developed early joint detectors that are tuned to all possible combinations of spatial and temporal frequency, orientation, disparity—as well as curvature—to solve the inverse problem of 3D motion (Lages et al., 2007; Lages and Heron, 2010). Instead the visual system may rely on binocular motion constraints of less specialized detectors in concert with binocular disparity input to capture non-linear 3D motion trajectories.

Early motion and disparity processing in the human visual system show tuning characteristics that supplement each other. Motion processing tends to have high temporal but relatively coarse spatial resolution whereas disparity processing has high spatial and relatively limited temporal resolution (Tyler, 1971; Lappin et al., 2009). It has been suggested that both, early motion and disparity detection, contribute to 3D motion perception and that this information is integrated late in the visual hierarchy (Likova and Tyler, 2007; Lages and Heron, 2008, 2010). At an early level of processing local motion detectors may encode motion constraints so that binocular motion processing remains ambiguous in terms of local motion direction but provides flexible spatio-temporal constraints (Lages and Heron, 2010). Disparity processing on the other hand offers fine spatial detail and depth information to disambiguate motion trajectories. Following the principle of least commitment (Marr, 1976) the two processing streams may define a characteristic spatio-temporal window for 3D motion perception (Tyler, 1971; Lages et al., 2003).

In an influential paper Weiss et al. (2002) demonstrated that 2D motion perception, modeled as a probabilistic inference, results in not necessarily veridical representations of physical stimulus motion (see also Ji and Fermuller, 2006). They proposed a Bayesian model of 2D motion perception that is based on likelihood constraints and a slow motion prior to explain a range of 2D motion illusions. Similarly, Lages (2006) suggested a Bayesian 3D motion model where binocular motion constraints can explain perceptual bias of horizontal motion trajectories in depth (Harris and Dean, 2003).

**Figure 1:**
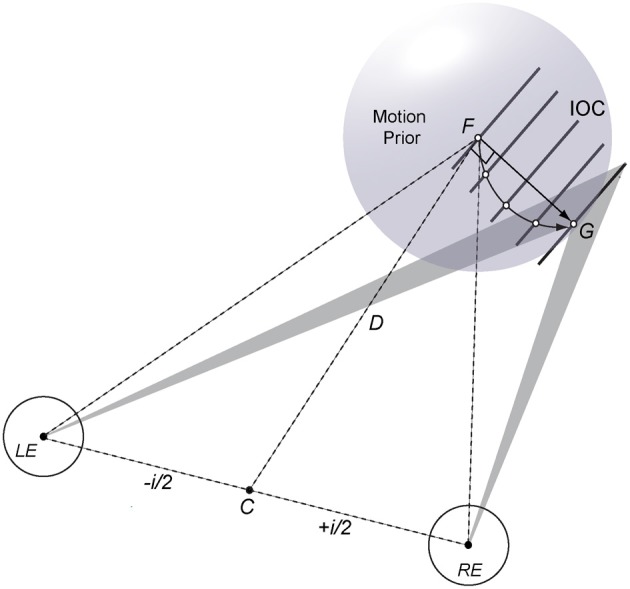
**Illustration of a geometric-statistical model for perceiving an oriented line or edge moving in 3D**. The left (LE) and right eye (RE), separated by interocular distance *i* are fixated on point *F* at distance *D*. If there are only velocity constraints (shaded triangles) a probabilistic 3D motion model estimates a linear trajectory (straight arrow from *F* to *G*) using a weak motion prior favoring slow motion (sphere centered on *F*). Binocular disparity processing provides additional information in form of intersections of constraints (IOCs; oriented lines) or texture elements (open dots) that help to disambiguate a concave motion path of the stimulus line in depth (curved arrow from *F* to *G*).

A geometric-statistical model based on motion and disparity constraints provides a flexible computational framework for binocular 3D motion perception that can capture arbitrary 3D trajectories of moving features and objects. For example, motion and disparity information needs to be combined to disambiguate local motion direction of a single line or edge moving in depth (see Figure 1 for an illustration). Uncertain and ambiguous motion input poses a particular problem for deterministic models because early encoding makes it difficult to explain perceptual bias and to adjust local motion vectors when additional information about the motion path becomes available (e.g., shading, angular size, texture, occlusion, endpoints). In a probabilistic approach binocular constraints can be established in terms of velocity and (dynamic) disparity likelihoods where updates of disparity constraints at intermediate positions help to disambiguate 3D motion trajectories. If the stimulus has unique features such as moving endpoints, corners, or texture elements then their trajectories can be “tracked” in depth over time. If, however, there are only uncertain or ambiguous moving features then a weak prior favoring slow motion (or short displacement in 3D space) suggests a linear trajectory as the default solution (see Figure 1; Lages et al., 2013). Such a prior may reflect tuning characteristics of a population of binocular motion cells and can explain perceptual bias. In general, the spatio-temporal features of a moving stimulus will influence how the 3D motion system combines and disambiguates motion and disparity information. Where exactly in the brain motion and disparity constraints are integrated is a matter of ongoing research (V3B/KO, hMT+/V5, occipital-temporal region; Likova and Tyler, 2007; Rokers et al., 2009; Ban et al., 2012). Importantly however, neural activation specific to motion and disparity processing seems to occur late rather than early in the visual hierarchy.

The observations by Pierce et al. (2013) demonstrate that binocular information processing facilitates discrimination of curved trajectories of a moving object. Perception of lines or contours of an object moving on a curved or non-linear path requires integration of binocular motion and disparity information. This is difficult to achieve in a deterministic framework where monocular motion vectors are linear, may not match up, and where disparity input indicates changed depth over time but no motion. In a probabilistic framework however, motion constraints can be combined with binocular disparity constraints and a motion prior to model the not necessarily veridical perception of curved trajectories.
